# Genome-wide identification and expression analysis of BZR gene family and associated responses to abiotic stresses in cucumber (*Cucumis sativus* L.)

**DOI:** 10.1186/s12870-023-04216-9

**Published:** 2023-04-24

**Authors:** Shilei Luo, Guobin Zhang, Zeyu Zhang, Zilong Wan, Zeci Liu, Jian Lv, Jihua Yu

**Affiliations:** 1 State Key Laboratory of Aridland Crop Science, Gansu Agricultural University, Lanzhou, China; 2 College of Horticulture, Gansu Agricultural University, Lanzhou, China

**Keywords:** BZR transcription factor, Genome-wide expression analysis, Abiotic stress

## Abstract

**Background:**

BRASSINAZOLE-RESISTANT (BZR) is a class of specific transcription factor (TFs) involved in brassinosteroid (BR) signal transduction. The regulatory mechanism of target genes mediated by BZR has become one of the key research areas in plant BR signaling networks. However, the functions of the BZR gene family in cucumber have not been well characterized.

**Results:**

In this study, six *CsBZR* gene family members were identified by analyzing the conserved domain of BES1 N in the cucumber genome. The size of CsBZR proteins ranges from 311 to 698 amino acids and are mostly located in the nucleus. Phylogenetic analysis divided *CsBZR* genes into three subgroups. The gene structure and conserved domain showed that the *BZR* genes domain in the same group was conserved. Cis-acting element analysis showed that cucumber *BZR* genes were mainly involved in hormone response, stress response and growth regulation. The qRT-PCR results also confirmed *CsBZR* response to hormones and abiotic stress.

**Conclusion:**

Collectively, the *CsBZR* gene is involved in regulating cucumber growth and development, particularly in hormone response and response to abiotic stress. These findings provide valuable information for understanding the structure and expression patterns of *BZR* genes.

**Supplementary Information:**

The online version contains supplementary material available at 10.1186/s12870-023-04216-9.

## Background

Brassinosteroids (BRs) are a growth-promoting steroid hormone found in addition to growth hormone (IAA), cytokinin (CTK), ethylene (ETH), gibberellin (GA), and abscisic acid (ABA) [1]. BR plays a vital role in regulating cell germination, cell elongation and division, leaf senescence, photomorphogenesis, pollen tube growth, flowering control, male reproduction, proton pump activity, nucleic acid and protein synthesis, gene expression, photosynthesis and other physiological processes [2–3]. After being accepted by the membrane kinases such as BRISSINOSTEROID INSENSITIVE 1 (BRI1) and BRI1-ASSIMILATED RECEPTOR KINASE1 (BAK1), BR activates the activity of the transcription factor BRI1 EMS SUPPRESSOR 1 (BES1) and its homologous protein BRISINAZOLE RESISTANT1 (BZR1) through signal transduction [4].

*BZR1* and *BES*1 belong to the same family of transcription factors, which play a key role in the BR signaling pathway in plants. The amino acid sequence similarity of BZR1 and BES1 is 88%, and the sequence consistency of the DNA binding domain is 97% [5]. Yin et al. further confirmed that *BES1/BZR1* is a specific class of transcription factors unique to plants and the only transcription factor of the BR signal transduction pathway [6]. The BZR protein contains a nuclear localization sequence (NLS) at the N-terminal, a highly conserved DNA binding domain, a phosphorylation domain (which can be phosphorylated by BRASSINOSTEROID INSENSITIVE2-BIN2), a PEST sequence and a C-terminal domain [6]. BR forms heterodimers by interacting with other regulatory proteins and binds to the BR response element (BRRE) or E-box in the promoter region of the target gene to regulate the expression of downstream BR response genes. Changes in the expression of downstream BR response genes will lead to a series of changes in physiology, biochemistry, growth and development of plants [7, 8]. In *Arabidopsis thaliana*, 953 BR regulated *BZR1* target genes (BRBT) and 1609 potential target genes of *BES1* were identified by transcriptional expression profile analysis and chromatin-immunoprecipitation microarray (ChIP) [9]. The activation or inhibition of *BZR1* and *BES1* on the target gene depends on the binding site or flanking sequence of the target gene promoter [10].

Currently, 8 *BZR/BES* gene family members have been identified in *Arabidopsis thaliana*, 6 in rice, 11 in maize, 15 in Chinese cabbage and 9 in tomato. Further experiments showed that *BZR/BES* gene family was involved in hormone signal transduction, regulation of growth and development and abiotic stress resistance [6, 11–16]. Research on BZR transcription factors in plant species has demonstrated their involvement in the regulation of cell elongation and division, plant morphology, flowering and fertility, quality improvement, and fruit ripening [17–25]. Cucumber (*Cucumis sativus* L.) is an important horticultural crop in the cucurbitaceae plant family, with significant agricultural, biological, and economic value worldwide [26, 27]. China is the largest cucumber producer in the world, accounting for about three quarters of the world’s total output every year [28]. Cucumber fruits are a rich source of various minerals, vitamins, carbohydrates, proteins, and dietary fiber and are also considered a natural antioxidant crop [29, 30]. Despite the known role of the *BZR/BE*S gene family in regulating plant growth and development through BR and other signaling pathways, the function of *BZR* genes in cucumber has not been reported yet. Therefore, in this study, we analyzed the *BZR* gene family in cucumber, including its chromosome distribution, gene structure, phylogenetic relationships, conserved sequences, and cis-acting elements. Additionally, we examined the expression pattern of *CsBZR* gene family members under different hormone and stress treatments. The findings of this study provide crucial information on the function and potential mechanisms of *BZR* family genes in cucumber.

## Results

### Identification and characterization of ***CsBZR*** gene family

**Fig. 1:**
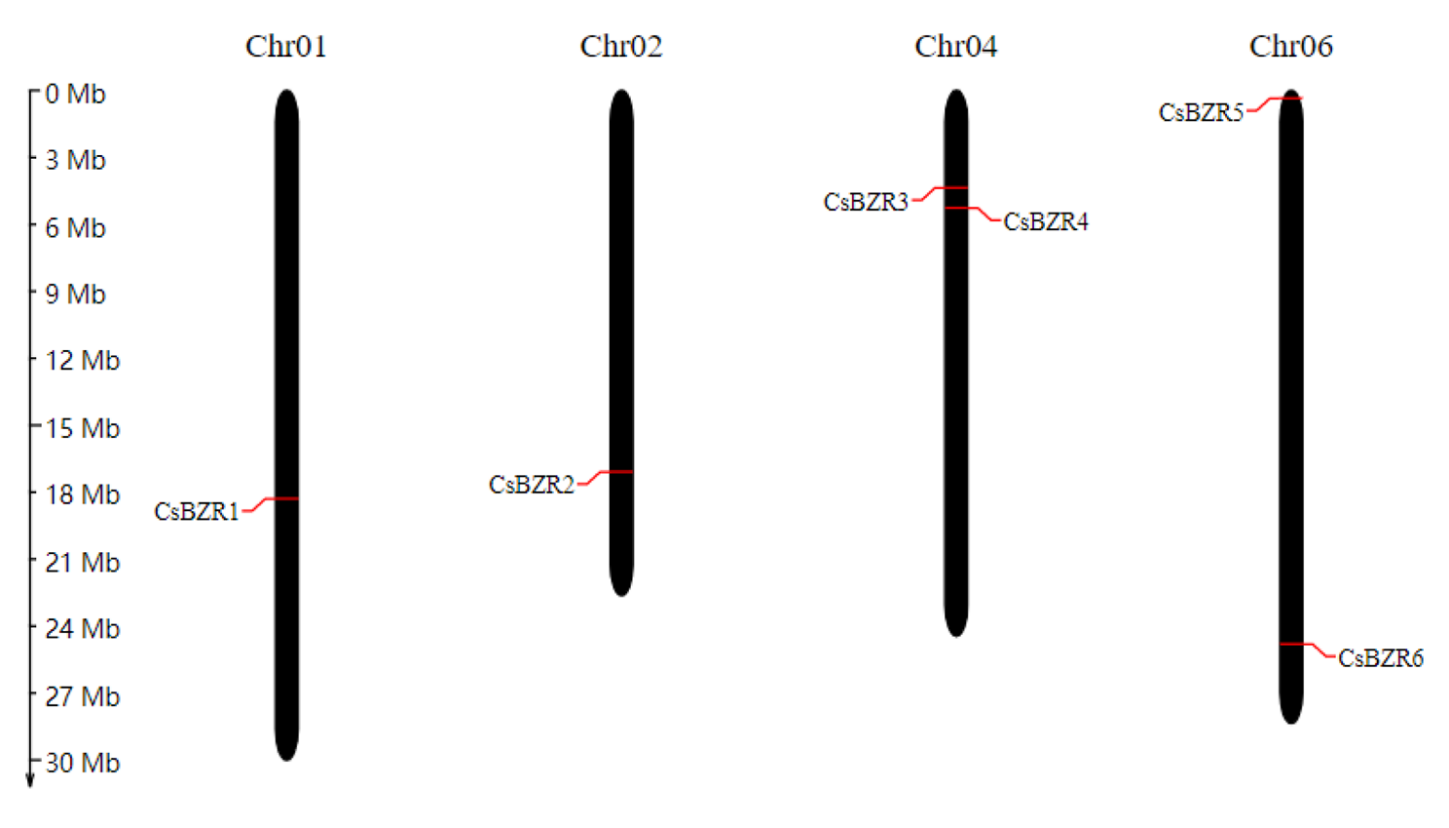
The distribution of *BZR* genes in cucumber chromosomes. Chromosome sizes can be estimated using the scale (in Megabases, Mb) to the left of the figure

Six candidate genes of *CsBZR* were identified using *AtBES* as seed sequence in the cucumber database, the same result was obtained through an online HMM search. Then the existence of the conserved BES1-type domain was verified by CD search and SMART. These 6 *CsBZR* genes were named *CsBZR1* to *CsBZR6* according to their location on the chromosome (Fig. 1). The *CsBZR* genes were distributed on four chromosomes of cucumber. *CsBZR1* and *CsBZR2* were located on chr01 and chr02, respectively. The chr04 contained *CsBZR3* and *CsBZR4*, while *CsBZR5* and *CsBZR6* were located at the head and tail of chromosome 6 respectively. It was worth noting that *CsBZR3* and *CsBZR5* were annotated as β-amylase, the rest were labeled as transcription factors. The predicted sizes of BZR family members in cucumber ranged from 311 to 698 amino acids, and their isoelectric points ranged from 5.78 to 9.71. Subcellular localization prediction showed that all *CsBZR* genes were located in the nucleus, which was consistent with the function of transcription factors (Table 1).

**Table 1:** *CsBZR* gene family and encoding protein properties

Gene name	Accession number	Chromosome location	AA	MW (kDa)	pI	Subcellular localization
*CsBZR1*	CsaV3_1G033010.1	chr1: 20,054,584 . 20,057,911	327	35.34	8.97	nucleus
*CsBZR2*	CsaV3_2G028510.1	chr2: 18,745,171 . 18,747,558	311	34.18	9.17	nucleus
*CsBZR3*	CsaV3_4G007080.1	chr4: 4,795,128 . 4,801,821	325	34.68	8.5	nucleus
*CsBZR4*	CsaV3_4G008150.1	chr4: 5,720,049 . 5,724,301	669	75.25	5.91	nucleus
*CsBZR5*	CsaV3_6G000500.1	chr6: 340,020 . 347,037	698	78.34	5.78	nucleus
*CsBZR6*	CsaV3_6G045980.1	chr6: 27,211,402 . 27,215,408	319	34.63	8.96	nucleus

AA: Number of amino acids; MW: Molecular weight; *pI*: Theoretical Isoelectric point

### Phylogenetic tree of ***BZR*** genes in cucumber

**Fig. 2:**
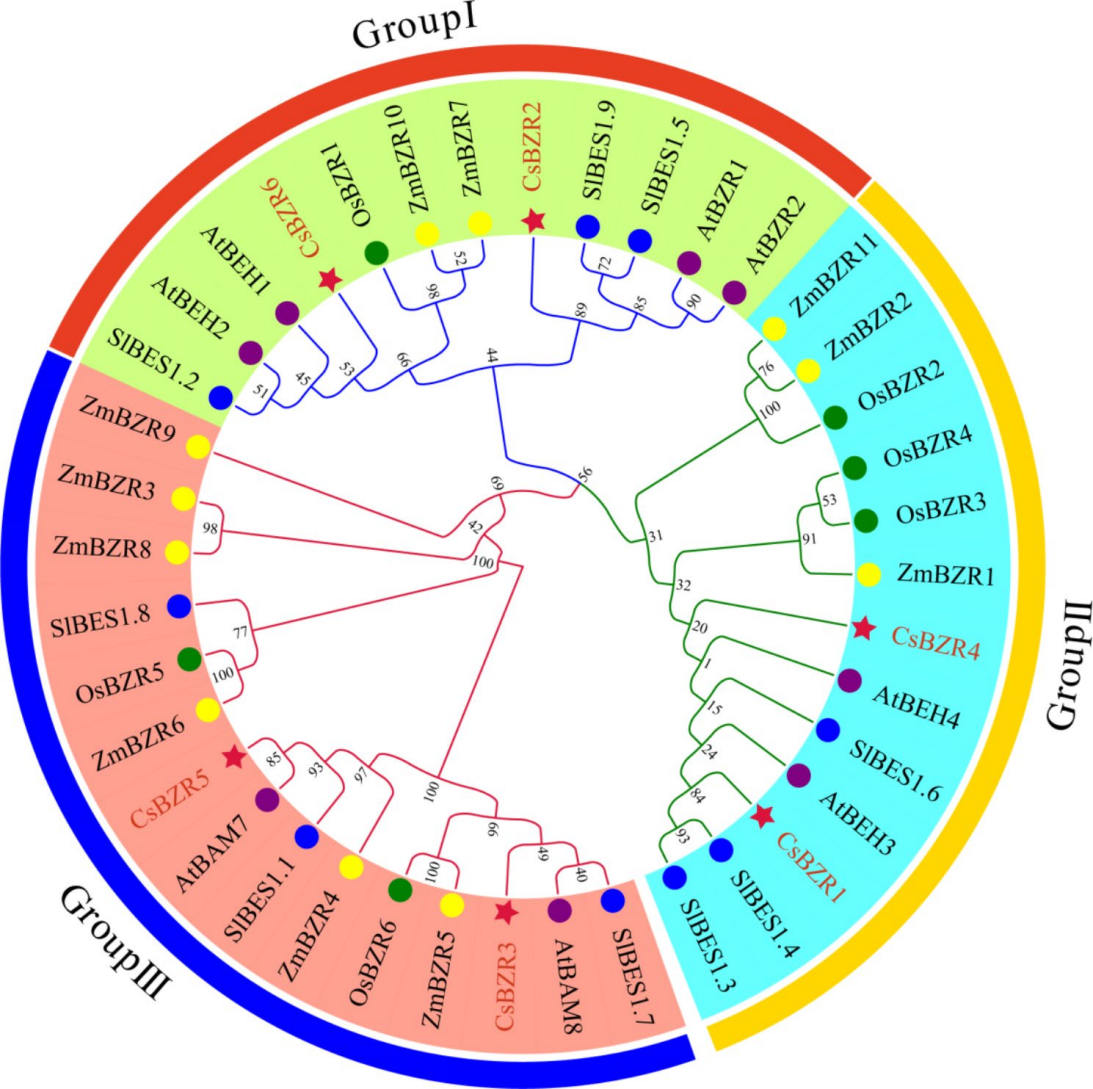
Phylogeny and collinearity analysis of CsBZR family. The phylogenetic tree consisted of five species, cucumber, *Arabidopsis*, tomato, corn and rice, and was divided into three subgroups. Different colors represent different groups. The number on each branch represents the self-expanding value

To further understand the phylogenetic mechanism of *CsBZR gene* family, the full-length protein sequences of BZR family in cucumber, *Arabidopsis*, rice, maize and tomato were used to construct phylogenetic trees (Fig. 2). We divided *BZR* genes into three groups based on their phylogenetic relationship, named Group I to Group III. The *AtBZR1* and *AtBZR2* as key members and their homologous genes *CsBZR2* and *CsBZR6* were classified as Group I. Furthermore, *OsBZR1* also appeared in Group I, indicating that they were evolutionarily and functionally conservative. Group II contained two members, *CsBZR1* and *CsBZR4* which belonged to the same subgroup as *AtBEH3*, *AtBEH4*, *SIBES1.3*, *SIBES1.4*, *SIBES1.6*, *OsBZR2*, *OsBZR3*, *OsBZR4*, *ZmBZR1*, *ZmBZR2* and *ZmBZR11*. Members of Group III had long amino acid sequences and were annotated as β-Amylase. The homologous genes of *AtBAM7* and *AtBAM8* were *CsBZR3* and *CsBZR5*, respectively.

### Gene structure and amino acids conserved motif of ***CsBZR*** genes

**Fig. 3:**
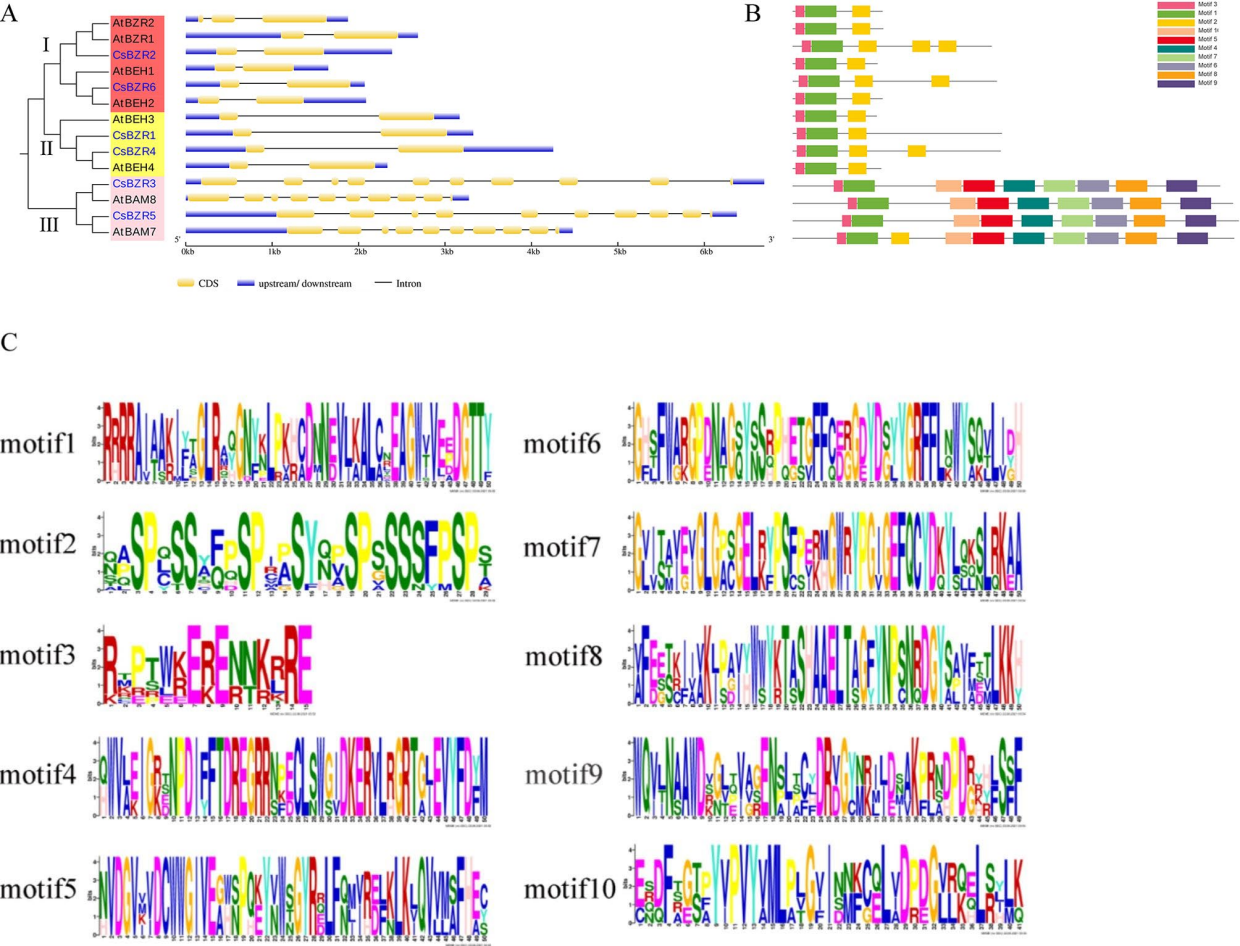
Structure and conserved motifs of *CsBZR* and *AtBES1* genes. Construction of phylogenetic tree using *Arabidopsis* and cucumber protein sequences (**A**). GSDS was used to visualize gene structure (**B**). The blue cells represent the untranslated region (UTR), the yellow units represent coding sequences (CDs), and the lines represent introns. MEME was used to analyze conserved motifs. Different color squares represent different motifs (**C**)

The phylogenetic tree constructed using CsBZR and AtBES1 protein sequences was divided into three groups (Fig. 3A), and the gene structure of the same group was very close. The genes in Group I contained 2–3 CDs and 1–2 introns (red region). All the genes in Group II had only one intron and two exons (yellow region). It was worth noting that the annotation was β-Amylase genes (*CsBZR3*, *CsBZR5*, *AtBAM7* and *AtBAM8*) all contain 10 exons, which were much more than other genes.

Highly similar amino acid sequences in functional domains often indicate similar biological functions, thus we explored 10 motifs of BZR proteins (Fig. 3C) in cucumber and *Arabidopsis*. As shown in Fig. 3B, motif 1 and motif 3 were the most conserved among all the genes. Annotation as β-Amylase genes *AtBAM7* in Group III contained motif 1 - motif 10, while the other genes contained other motifs except motif 2, while Group I and Group II only contained motif 1, motif 3 and motif 2. The same subgroup consists of almost the same conserved motifs, indicating that they may have similar functions.

### Collinearity analysis of BZR family genes in cucumber

**Fig. 4:**
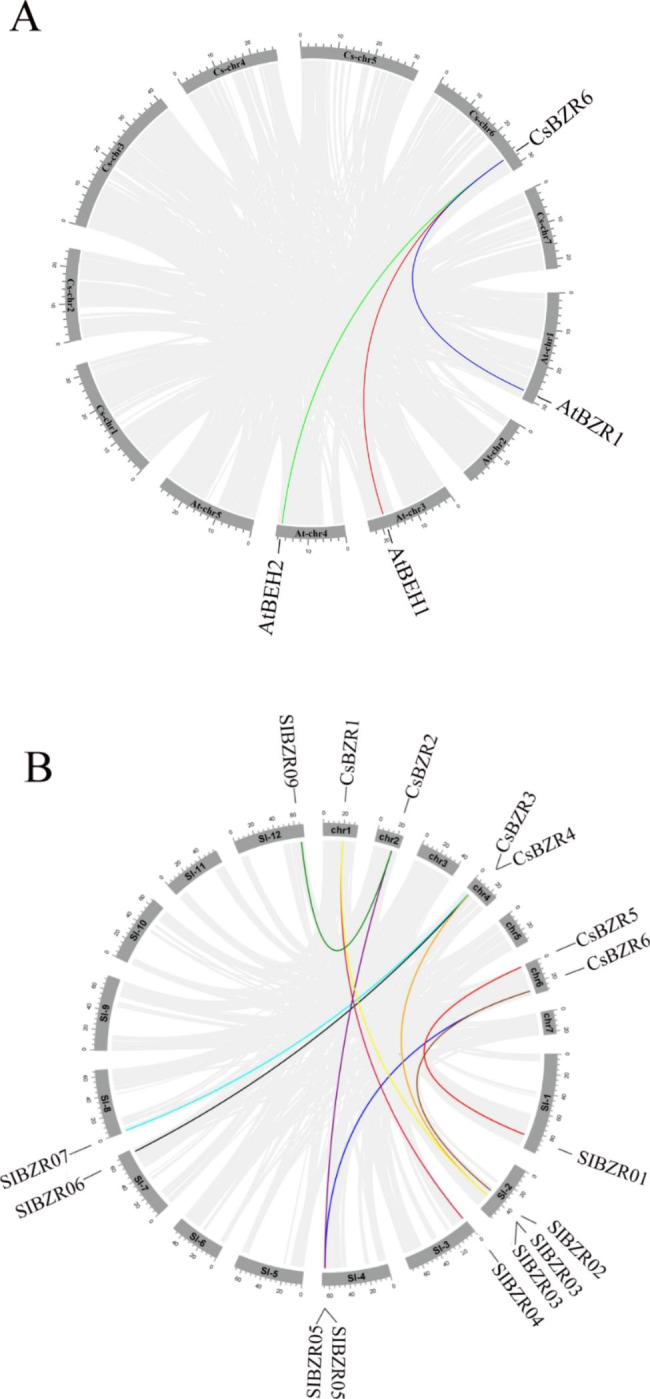
Collinearity of the orthologous *BZR* genes in (**A**) *Arabidopsis*, (**B**) tomato and cucumber. The lines with different colors represent gene pairs. The collinear relationship of all orthologous genes in different species was shown with the gray lines

No collinear gene pairs were found in cucumber species, while there were more collinear gene pairs in tomato and cucumber than in *Arabidopsis* and cucumber. Collinearity analysis showed that *CsBZR6* had collinear gene pairs with *AtBZR1*, *AtBEH1*, and *AtBEH2* in *Arabidopsis* and cucumber (Fig. 4A). All *CsBZR* genes were found to have a collinear relationship with *SlBZR* genes, resulting in a total of 10 pairs (Fig. 4B). These results illustrated that the genetic relationship between *CsBZR* genes and *SlBZR* genes was close.

### Analysis of cis acting elements in ***CsBZR*** genes

**Fig. 5:**
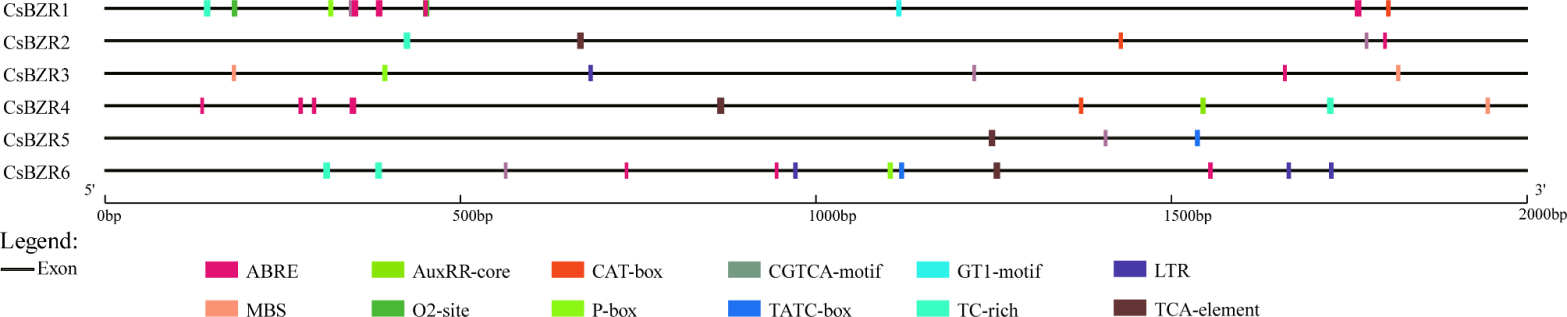
Cis-element analysis in the promoters of *CsBZR* genes. The above figure shows the position of cis-elements at 2 kb upstream of *CsBZR* gene. Different color squares represent different elements

Cis-element prediction can help further understand the potential regulatory mechanism of *CsBZR* genes. The upstream promoter region of cucumber BZR family genes contained many cis-elements (Fig. 5), that could respond to plant hormones, abiotic stresses and plant development-related (Table 2). Among these cis-elements, ABRE, AuxRR-core, CGTCA-motif, P-box, TATC-box, TGACG-motif and TCA-element were involved in abscisic acid, auxin, methyl jasmonate, gibberellin and salicylic acid responsiveness, respectively. In addition, *CsBZR* genes were also involved in low-temperature, drought and meristem expression. These results indicated that *CsBZR* family genes may be involved in variety of stress and plant hormone response processes, which could effectively promote plant growth and stress resistance, as well as have important biological functions.

**Table 2:** Functional annotation of cis-elements

Response process	Element name	Functional annotation
Hormone response	ABRE	cis-acting element involved in the abscisic acid responsiveness
	AuxRR-core	cis-acting regulatory element involved in auxin responsiveness
	CGTCA-motif	cis-acting regulatory element involved in the MeJA-responsiveness
	P-box	gibberellin-responsive element
	TATC-box	cis-acting element involved in gibberellin-responsiveness
	TGACG-motif	cis-acting regulatory element involved in the MeJA-responsiveness
	TCA-element	cis-acting element involved in salicylic acid responsiveness
Stress response	LTR	cis-acting element involved in low-temperature responsiveness
	MBS	MYB binding site involved in drought-inducibility
	TC-rich repeats	cis-acting element involved in defense and stress responsiveness
Plant development response	GT1-motif	light responsive element
	O2-site	cis-acting regulatory element involved in zein metabolism regulation
	CAT-box	cis-acting regulatory element related to meristem expression

### Tissue specific expression pattern of ***CsBZR*** genes

**Fig. 6:**
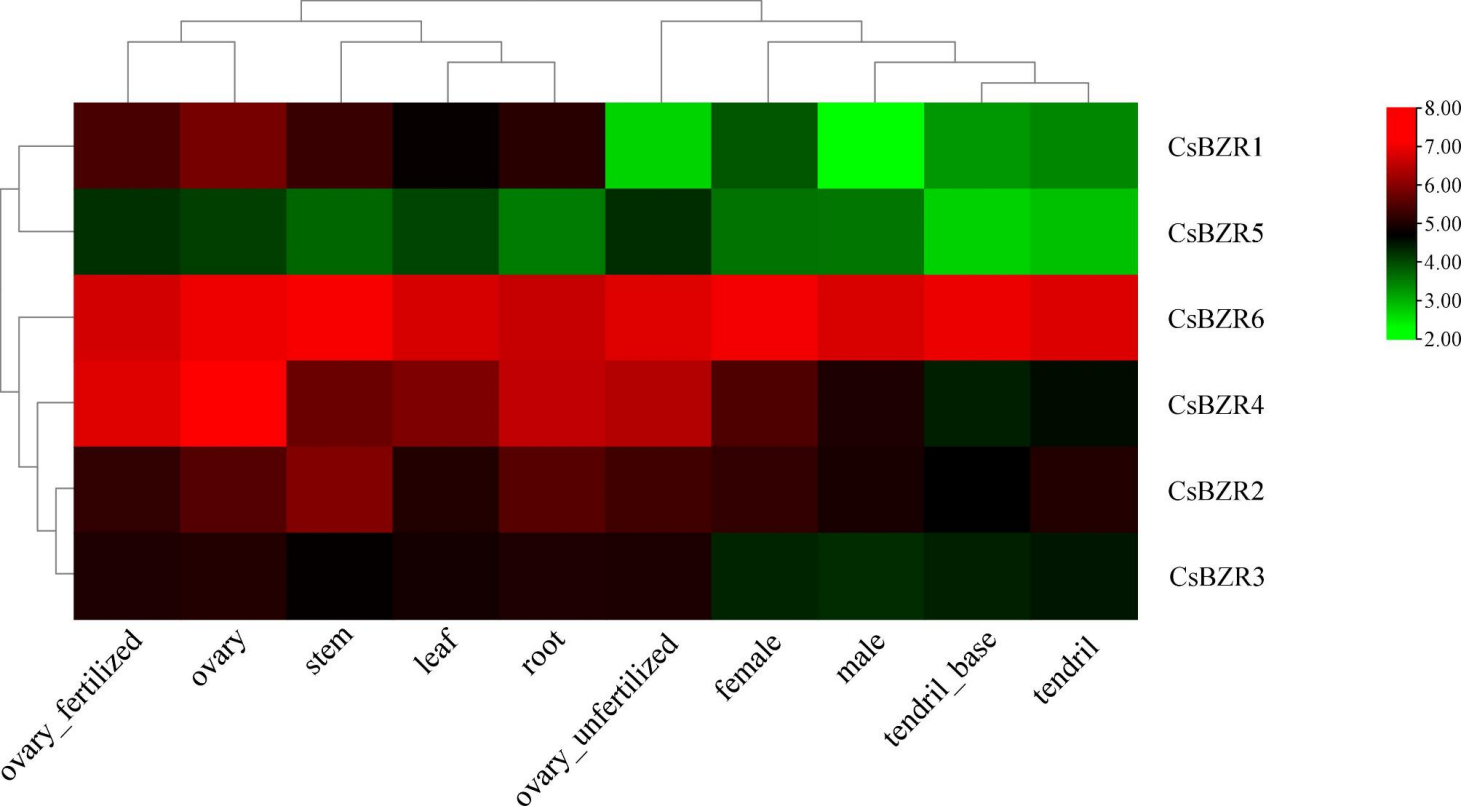
Expression patterns of *CsBZR* genes in different tissues. The tissue expression data were obtained from cucumber database, and the heat map was made by TBtools software. The color scale indicates the level of gene expression

In order to explore the expression of *CsBZR* gene in different tissues of cucumber, we downloaded relevant data from cucumber genome website and used TBtools software to create an expression pattern heat map. As shown in Fig. 6, *CsBZR6* was widely expressed in all tissues and organs and showed high expression levels, indicating that *CsBZR6* played a vital role in cucumber tissue development. Conversely, the expression levels of *CsBZR5* were low in all tissues. The expression level of *CsBZR4* in ovary_fertilized, ovary, ovary_unfertilized and roots was also at a high level, while was the low expression in tendril_base and tendril.

### Expression profiles of ***CsBZR*** genes in response to plant hormone

**Fig. 7:**
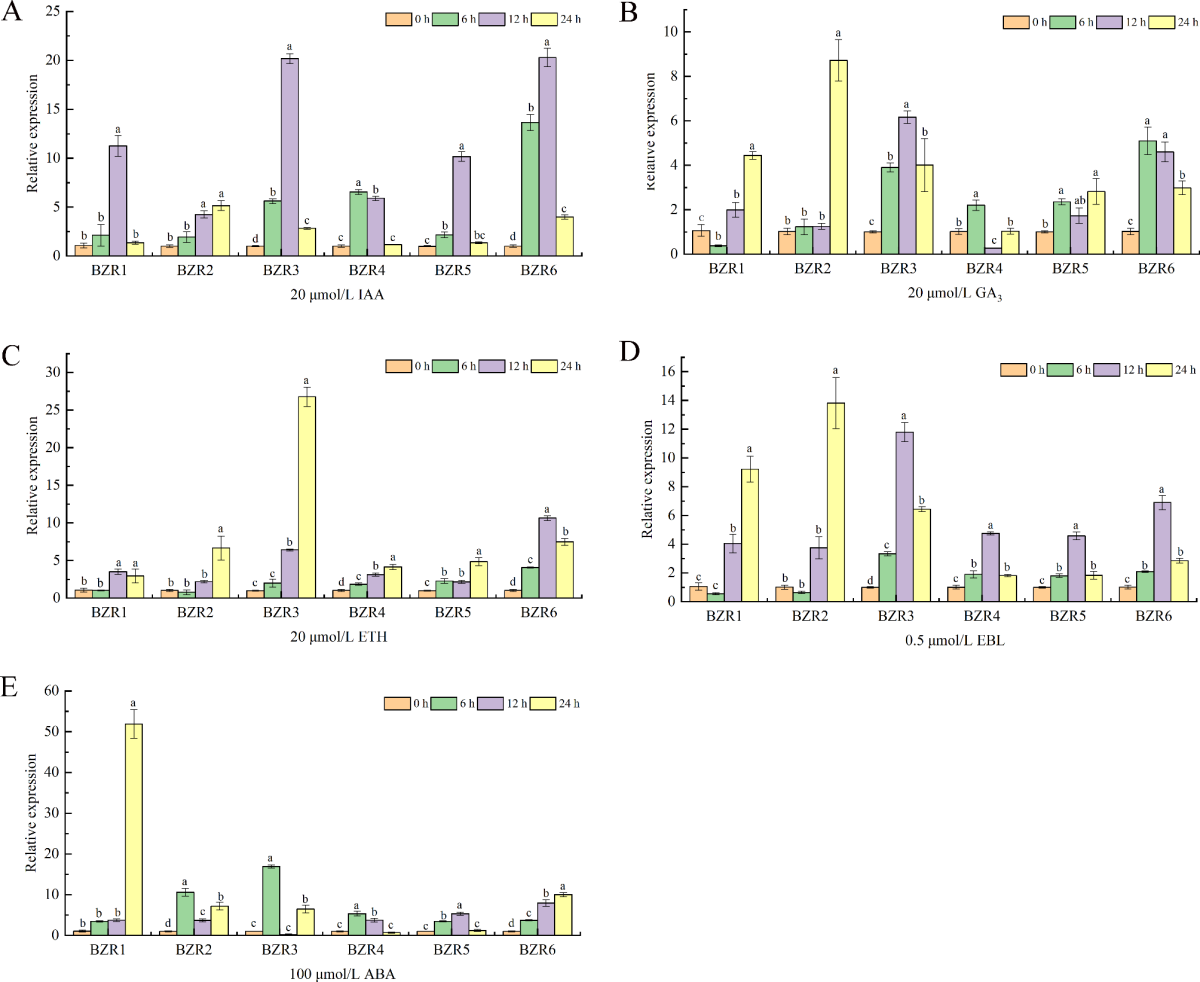
*CsBZR* genes expression patterns under different hormone treatments. (**A**) 200 µM IAA, (**B**) 20 µM GA3, (**C**) 20 µM ETH, (**D**) 0.5 µM EBL and (**E**) 100 µM ABA were used to treat cucumber seedlings and sampled at 0, 6, 12 and 24 h, respectively. The data are means ± SE of three biological replicates. Different letters indicate significant differences (*P* < 0.05; Duncan’s multiple range tests)

BZR transcription factors are usually reported to be involved in plant growth and development. The indole-3-acetic acid (IAA), Gibberellin A3 (GA3), Abscisic Acid (ABA), ethephon (ETH), epi-brassinolide (EBL) were used to detect the response of *CsBZR* genes. As shown in Fig. 7, all *CsBZR* genes significantly responded to IAA, with the relative expression of *CsBZR3* and *CsBZR6* at 12 h being 20-fold higher than at 0 h. With the exception of *CsBZR4* and *CsBZR5*, the response of the other *CsBZR* genes to GA3 was over four times greater. *CsBZR3* showed the most significant response to ETH, with a relative expression of 26-fold greater than at 0 h, while the relative expression of other *CsBZR* genes ranged from 2 to 7. *CsBZR* genes also significantly responded to EBL, with the relative expression of *CsBZR1*, *2*, and *3* being 9, 13, and 11-fold greater than at 0 h, respectively. Similar to EBL treatment, ABA induced up-regulation of all *CsBZR* genes, with relative expression multiples of 51, 10, 16, 5, 5, and 10 times, respectively. Overall, these results suggested that *CsBZR* genes may be involved in various hormone regulations in a complex manner, and the crosstalk between this family and plant hormones needs to be explored in detail.

### Expression profiles of ***CsBZR*** genes in response to abiotic stress

**Fig. 8:**
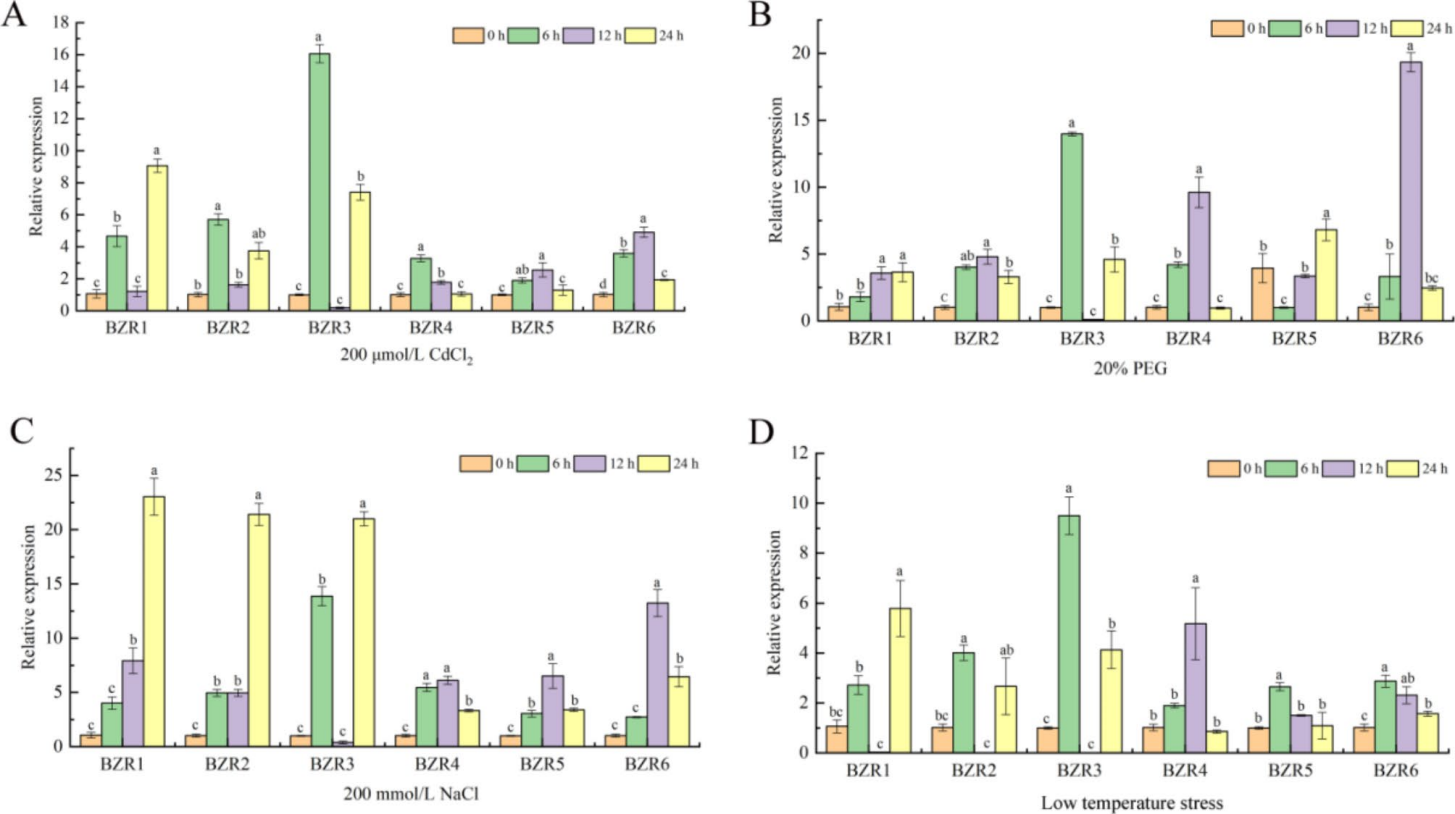
*CsBZR* genes expression patterns under different stresses. (**A**) 200 µM CdCl_2_, (**B**) 20% PEG, (**C**) 200 mM NaCl and (**D**) low temperature were used to treat cucumber seedlings and sampled at 0, 6, 12 and 24 h, respectively. The data are means ± SE of three biological replicates. Different letters indicate significant differences (*P* < 0.05; Duncan’s multiple range tests)

In order to explore the response of *CsBZR* genes to abiotic stress, we verified their expression patterns under heavy metals, drought, salt and low temperature. As shown in Fig. 8, the relative expressions of *CsBZR4* and *CsBZR5* under Cd stress were low, ranging from 2 to 3, while the relative expressions of *CsBZR1*, *CsBZR2*, *CsBZR3* and *CsBZR6* were high, which were 4, 5, 16 and 5 folds higher than that of 0 h, respectively. Under 20% PEG treatment, the relative expression of all *CsBZR* genes was greater than 3, and the responses of *CsBZR3* and *CsBZR6* were particularly significant, reaching 13.9 and 19.3. Salt stress induced up regulation of all *CsBZR* genes, in which *CsBZR1*, *2*, *3* had a high expression. The response expression of *CsBZR1*, *2*, *3*, *4* to low temperature stress was higher than 4, while the response expression of *CsBZR5*, *6* was lower than 3. The above results showed that *CsBZR* genes could respond to a variety of abiotic stresses and were up-regulated.

## Discussion

BZR protein is an important class of transcription factors in plants, and it is also a key component of BR signal transduction pathway. BZR protein not only regulates plant growth and development, but also participates in the response of many plants to abiotic stress [15, 31–34]. Since the *BZR* gene family of *Arabidopsis thaliana* was excavated, *BZR* gene family of many other species have also been identified, including *Brassica rapa* [15]、*Beta vulgaris* [35]、*Zea mays* [14]、*Triticum aestivum* [36] and tomato [12]. Characterization and systematic analysis of BZRs in cucumber have not been completed to date. In this study, we identified the members of cucumber BZR family, and analyzed the physical and chemical properties of the protein, gene structure, conservation, cis-elements and related expression patterns.

Six *BZR* family genes were identified from the whole genome of cucumber (Table 1). The number of cucumber BZR family members is two less than that of *Arabidopsis* and three less than that of tomato [12]. In addition to the 6 *BZR* genes initially discovered in *Arabidopsis*, it was later discovered that *BAM7* and *BAM8* in the BAM family also have a BRASSINAZOLE RESISTANT1 (BZR1) type DNA binding domain, which can also be found in transcription factors that mediate the brassinosteroid (BR) response [37]. In order to better understand the evolutionary relationship among *BZR* family members in different species, we constructed a phylogenetic tree with BZR proteins from cucumber, *Arabidopsis*, tomato, maize and rice. Phylogenetic analysis showed that *CsBZR* genes were divided into three subfamilies. The first subgroup included *AtBZR1* and *OsBZR1*, and *CsBZR2* and *CsBZR6* belong to the same group. The second subgroup contained *CsBZR1* and *CsBZR4*, which are close to the branches of *SiBES1.3*, *SiBES1.4* and *SiBES1.6*. The third subgroup contained *CsBZR3* and *CsBZR5*, which are annotated as starch hydrolases together with *AtBAM7* and *AtBAM8*. Tandem repeat and fragment replication is the main ways of gene family replication, and participate in the formation and functional differentiation of new genes [38]. In our study, there was no collinear relationship between cucumber *BZR* genes, while *CsBZR6* and *AtBZR1*, *AtBEH1* and *AtBEH2* had collinearity. All cucumber *BZR* genes and tomato *BZR* genes form collinear pairs, indicating that they are more closely related genetically.

In terms of gene structure and conserved motifs, the classification of *Arabidopsis* and cucumber is similar to that of the evolutionary tree. *CsBZR3*, *CsBZR5*, *AtBAM7* and *AtBAM8* annotated as starch hydrolases were divided into the same group, and there were significant differences in structure and conserved motifs from other *BZR* genes. In *Arabidopsis*, *BAM7* and *BAM8* have a BAM-like domain. In addition to the function of normal BZR protein, BZR1-BAMS can regulate many genes responding to BRs, and also regulate by binding the ligands in the BAM-like domain to transmit metabolic signals, plant growth and development can be controlled by crosstalk with BR signals [37, 39]. Compared to other *BZR* genes, *CsBZR3* and *CsBZR5* have a greater number of introns, exons and conserved motifs diversity, suggesting that they may have additional functions, such as involvement in amylase hydrolysis. These results showed that most *BZR* genes come from the same ancestor and their functions are conserved.

Cis-acting elements are noncoding DNA sequences that exist in the promoter region and participate in the regulation of gene expression. In this study, we selected 12 elements and divided them into three categories (hormone response, stress response and plant development response). Hormone response included responses to ABA, IAA, MeJA, gibberellin and salicylic acid. Stress responses included low-temperature, drought-inducibility and defense and stress responsiveness. Plant development included light responsive elements, zein metabolism regulation and meristem expression. Some reports also confirmed that the BZR family is involved in a variety of hormone signaling pathways [40–42], stress response [15, 43–45], and plant growth regulation [19, 46, 47].

BZR family genes are specifically expressed in different tissues and organs of many plants [14, 16, 44]. In *Arabidopsis*, the transcriptional expression level of *BZR* gene was higher in roots and buds, but lower in stems, fruits and flowers [48, 49]. The expression level of BZR family genes was high in maize seedlings and endosperm [14]. *SlBZR02* and *SlBZR09* genes in tomato are generally expressed in most tissues, and their expression levels are very high, while other *SlBZR* genes are only highly expressed in some tissues [16]. We used the published cucumber transcriptome data to analyze the expression pattern of *CsBZR* genes in different tissues (root, stem, leaf, female, male, ovary_fertilized, ovary_unfertilized, tendril_base and tendril). *CsBZR6* was highly expressed in almost all tissues, while the transcriptional level of *CsBZR5* was relatively low. The remaining *CsBZR1*, *2*, *3* and *4* have a considerable transcriptional level in different tissues. Therefore, the differential expressions of *CsBZR* family members in different tissues indicate that they play a regulatory role in the growth and development of cucumber, and there is functional redundancy.

A large number of studies have shown that *BZR* gene is involved in the response to various signals, including IAA, ABA, GA, ETH, NaCl and drought stress [31, 42, 44, 50–52]. In the current study, we identified three major types of acting elements: hormone response, stress response and plant development response. The hormone response elements included ABA, IAA, methyl jasmonate, GA and salicylic acid responsiveness. The plant hormone response pattern verified that CsBZR family genes could be up-regulated at a variety of hormone levels, especially under ABA, ETH and EBR treatments (Fig. 7). BZR as a key transcription factor in BR signal transduction is involved in regulating multiple stress responses. Cis-acting element analysis showed that *CsBZR* genes were associated with low-temperature and drought. Under low-temperature treatment, the expression of *CsBZR1*, *3* and *4* were more than 4 folds compared to 0 h, while *CsBZR3*, *4*, *5* and *6* were significantly up-regulated under 20% PEG treatment. Studies in *Arabidopsis* showed that continuous low temperature treatment could accumulate dephosphorylated BZR1, and the frost resistance of functional acquired mutants of *BZR1* and *BES1* was significantly enhanced [45]. In Chinese cabbage, *BrBZR1-1*, *BrBES1-3*, *BrBEH4*, *BrBEH6*, *BrBEH7* and *BrBEH8* showed obvious responses to cold. These *BrBZR* transcription factors were considered to be the transcriptional activators of CBF cold response pathway in Chinese cabbage [15]. The findings of Ye et al. proved that the members of BES/BZR family in *Arabidopsis* played a role in response to drought stress. BES1 protein could directly and negatively regulate the expression of drought response gene *RD26*, thus mediating the signal cross between BR and drought pathway [53]. In wheat, the BES/BZR family transcription factor *TaBZR2* plays an active role in drought response and reduces the accumulation of reactive oxygen species by activating *TaGST1* [44]. Surprisingly *CsBZR6* was very active in the expression pattern in different tissues, while the expression was relatively low in hormone-induced and drought stresses. This could be because CsBZR6 was closely related to cucumber growth and development but was insensitive to hormone treatment and drought stress. *CsBZR* family genes responded significantly to salt stress, especially the expression of *CsBZR1*, *2* and *3* was more than 20-folds that of the 0 h. Similar results were also reported in maize and *Eucalyptus grandis* [14, 54]. In this study, we also analyzed the expression pattern of *CsBZR* genes under cadmium stress. Except *CsBZR4* and *CsBZR5*, the expression of other genes had a high level. Many studies have shown that BR can alleviate heavy metal stress and improve plant tolerance [55–57]. BZR, as the only transcription factor in BR signal, may have a potential regulatory effect under heavy metal stress. These findings reveal the complexity of cucumber *BZR* genes regulation and establish the foundation for further study on the function of the cucumber *BZR* genes.

## Conclusions

In this study, six cucumber *BZR* genes were identified, which were divided into three branches according to the systematic evolutionary tree. As key transcription factors in the BR signaling pathway, *CsBZRs* were involved in hormone and stress responses as well as growth regulation in cucumber. Tissue expression patterns showed that *CsBZR6* was highly expressed in various cucumber tissues and may be an important gene regulating cucumber growth and development. *CsBZRs* responded to hormone induction, particularly *CsBZR3* and *CsBZR6*. *CsBZRs* also participated in the regulation of abiotic stress, with *CsBZR1*, *CsBZR2* and *CsBZR3* showing significant responses to most stresses. These findings provide a reference for the future research on the function of *CsBZR* genes and the exploration of potential regulatory network.

## Materials and methods

### Plant material culture

Cucumber seeds (*Cucumis sativus* L., ‘xinchun 4’) were procured from the Gansu Academy of Agricultural Sciences, China. The seeds were kept in a dark environment at a temperature of 28℃ for the purpose of germination. After successful germination, the incubator conditions were adjusted to maintain a temperature of 25℃/18℃, with a photoperiod of 14/10 h, to ensure optimal growth of the seedlings.

Following 7 d of cultivation, the seedlings were transplanted into a plastic box containing the Yamazaki nutrient solution, for further cultivation. Based on the growth rate of the cucumber seedlings, the Yamazaki nutrient solution was varied in concentration, ranging from 1/8, 1/6, 1/4, 1/2, to 1. Subsequently, the seedlings were subjected to different treatments after 20 d of growth.

### Identification of ***BZR*** genes in cucumber

We performed a BLASTP search using the BZR gene family from *Arabidopsis* (https://www.arabidopsis.org/) in the cucumber genome database (http://www.cucurbitgenomics.org/) with an e-value of 10^− 10^. In addition, we downloaded the BES1 domain file (PF05687) from the Pfam website (http://pfam.xfam.org/) and uploaded it to the HMM website (https://www.ebi.ac.uk/Tools/hmmer/search/hmmsearch) to search for potential genes in the cucumber genome containing this conserved domain [58]. Duplicate genes were removed by comparing the results of the two different searches. The remaining genes were further validated for the presence of the BES1-type domain using the CD search website and the SMART website (http://smart.embl-heidelberg.de/). The *CsBZR* gene and protein sequence information can be found in Supplementary Material 1.

### Chromosome distribution Bioinformatic analyses of ***BZR*** gene family in cucumber

The position information of *CsBZR* family genes was retrieved from the cucumber genome database, and the distribution of genes on chromosomes was visualized using an online website (http://www.mg2c.iask.in/mg2c_v2.0/). The secondary structure and subcellular localization of the protein sequences were predicted using the ExPASy online website (https://web.expasy.org/protparam/).[59].

### Construction of phylogenetic tree and collinearity analysis

The phylogenetic tree of *BZR* gene family of cucumber, *A. thaliana*, tomato, corn, and rice were constructed by using MEGA 7 software with MUSCLE methods to align multiple sequences and bootstrapping was performed 1000 times to obtain self-expanding values for each branch in Fig. [60]. Finally, the neighbor-joining is selected to represent their evolutionary relationship and beautify the constructed tree by the EvolView website. See supplementary material 2 for registration numbers of various species.

McScanX was utilized to search for homology by comparing the protein-coding genes from the cucumber genome against the genomes of *Arabidopsis* and tomato using BLASTP, with a retrieval threshold set at E-value < E^− 5^. Default parameters were used for all other settings. Whole-genome BLASTP results were used to compute collinear blocks for all possible pairs of chromosomes and scaffolds [61]. Subsequently, TBtools was used to visualize the identified collinear pairs of cucumber, *Arabidopsis* and tomato [62].

### Gene structure and conservative motif analysis

The CDs sequence and genomic sequences were input into GSDS website (http://www.gsds.gao-lab.org/) for analysis, and the display map of intron/exon was obtained [63].

To analyze conservative motifs, the protein sequences of CsBZR and AtBZR were uploaded to the MEME website (http://meme-suite.org/tools/meme). The maximum number of motifs was set to 10, and all other parameters were set to their default values [64].

### Analysis of cis-acting elements in *CsBZR* gene promoters

TBtools software intercepted a 2000 bp region upstream of the start codon of *CsBZR* family genes as a promoter, and then the cis acting elements are predicted through PlantCare website (http://bioinformatics.psb.ugent.be/webtools/plantcare/html/) [65].

### Expression patterns of ***BZR*** gene family in different tissues of cucumber

CsBZR transcriptome data (FPKM) was downloaded from cucumber genome database (http://www.cucurbitgenomics.org/rnaseq/cu/3, BioProject PRJNA80169). Transcriptome data were normalized by logarithms, and then expression heat map were drawn using TBtools software. The color scale indicates the level of gene expression [66].

### Hormone and abiotic stress treatments

The expression pattern experiment was divided into hormone and abiotic stress treatments. To prepare the treatment concentrations, ABA, IAA, GA, and ETH were dissolved in 100 mL distilled water to a concentration of 1 mM, and then diluted accordingly. EBL was dissolved in 100 mL 90% ethanol solution to a concentration of 1 mM, and then diluted to the treatment concentration. Hormone treatments involved the application of ABA (100 µM), IAA (20 µM), GA (20 µM), EBL (0.5 µM), and ETH (20 µM). Samples were collected at 0, 6, 12, and 24 h post-treatment, and untreated seedlings from the same batch were used as controls. At each time point, three biological replicates were collected, with each replicate consisting of three independent seedlings.

Drought, low temperature, CdCl_2_ and salt were set as abiotic stress treatment. All reagents are purchased from Shanghai MACKLIN Co., Ltd. Add PEG 6000, CdCl_2_ and NaCl into 1 L nutrient solution to prepare 20% PEG 6000, 200 µM CdCl_2_ and 200 mM NaCl solution, respectively. The low temperature stress was set at 12℃/8℃. Samples were collected after treatment for 0, 6, 12 and 24 h respectively. Normal growth seedlings were used as control. Three individual seedlings were collected for one repetition and three biological replicates were collected at each time point.

### RNA isolation, cDNA synthesis and quantitative real-time PCR analysis

Total RNA was extracted using the DP432 kit (Tiangen, China) following the manufacturer’s instructions. cDNA was synthesized using the fastking cDNA dispersion RT supermax kit (Tiangen, China) with 2 µL of RNA as the template. The SYBR Green kit (Tiangen, China) was used for the fluorescence quantitative system. Primers were synthesized by Shanghai Shenggong Company. The reaction system volume was 20 µL, which contained 2 µL of cDNA solution, 10 µL of 2*SuperReal PreMix Plus, 0.6 µL of 10 µM forward and reverse primers, 0.4 µL of 50*ROX Reference Dye, and 6.4 µL of distilled deionized water. qRT-PCR analysis was performed using the LightCycler® 480 II real-time fluorescence quantitative PCR instrument. The reference gene used was Actin3 (DQ115883). The relative level of gene expression was analyzed using the 2^−∆∆Ct^ method. See supplementary material 3 for primers information.

## Electronic supplementary material

Below is the link to the electronic supplementary material.

Supplementary Material 1

Supplementary Material 2

Supplementary Material 3

Supplementary Material 4

## Data Availability

The qRT-PCR data supporting the gene relative expression results of this study can be found in supplementary material 4. The login numbers of all *CsBZR* genes identifed and tissue specific expression transcriptome data of *CsBZR* genes (BioProject PRJNA80169) can be obtained in the cucumber genome database (http://www.cucurbitgenomics.org).

## Abbreviations

BZR: BRASSINAZOLE-RESISTANT
BRI1: BRISSINOSTEROID INCENSIVE1
BAK1: BRI1-ASSIMILATED RECEPTOR KINASE1
BIN2: BRASSINOSTEROID INSENSITIVE2
NLS: N-terminal nuclear localization sequence
ChIP: Chromatin-immunoprecipitation microarray
IAA: Auxin
CTK: Cytokinin
ETH: Ethylene
GA: Gibberellins
ABA: Abscisic acid
MeJA: Methyl jasmonate
